# Diagnostic and Therapeutic Strategies for Stable Coronary Artery Disease Following the ISCHEMIA Trial

**DOI:** 10.1016/j.jacasi.2022.10.013

**Published:** 2023-02-15

**Authors:** Shun Kohsaka, Kentaro Ejiri, Hidenobu Takagi, Ippei Watanabe, Yodo Gatate, Kenji Fukushima, Shintaro Nakano, Taishi Hirai

**Affiliations:** a Department of Cardiology, Keio University School of Medicine, Tokyo, Japan; b Department of Epidemiology, Johns Hopkins Bloomberg School of Public Health, Baltimore, Maryland, USA; c Department of Diagnostic Radiology, Tohoku University Hospital, Sendai, Japan; d Department of Internal Medicine, Toho University Faculty of Medicine, Tokyo, Japan; e Department of Cardiology, Self-Defense Forces Central Hospital, Tokyo, Japan; f Department of Radiology, Fukushima Medical University, Fukushima, Japan; g Department of Cardiology, Saitama Medical University International Medical Center, Hidaka, Japan; h Department of Cardiology, University of Missouri, Columbia, Missouri, USA

**Keywords:** noninvasive testing, optimal medical therapy, pretest probability, revascularization, stable coronary artery disease, CABG, coronary artery bypass grafting, CAD, coronary artery disease, CTA, computed tomographic angiography, DAPT, dual antiplatelet therapy, EF, ejection fraction, FFR, fractional flow reserve, ICA, invasive coronary angiography, IVUS, intravascular ultrasound, LVEF, left ventricular ejection fraction, OMT, optimal medical therapy, OCT, optical coherent tomography, PCI, percutaneous coronary intervention, PTP, pretest probability, RCT, randomized clinical trial

## Abstract

Until recently, coronary revascularization with coronary artery bypass grafting or percutaneous coronary intervention has been regarded as the standard choice for stable coronary artery disease (CAD), particularly for patients with a significant burden of ischemia. However, in conjunction with remarkable advances in adjunctive medical therapy and a deeper understanding of its long-term prognosis from recent large-scale clinical trials, including ISCHEMIA (International Study of Comparative Health Effectiveness With Medical and Invasive Approaches), the approach to stable CAD has changed drastically. Although the updated evidence from recent randomized clinical trials will likely modify the recommendations for future clinical practice guidelines, there are still unresolved and unmet issues in Asia, where prevalence and practice patterns are markedly different from those in Western countries. Herein, the authors discuss perspectives on: 1) assessing the diagnostic probability of patients with stable CAD; 2) application of noninvasive imaging tests; 3) initiation and titration of medical therapy; and 4) evolution of revascularization procedures in the modern era.

## Highlights

• With advancements in its management, outcomes in stable CAD have progressively improved.
• However, the prevalence for stable CAD differs in Asian countries, and unmet needs exist between guideline recommendations and its actual clinical application.
• Decision making of stable CAD should be tailored for each patient, and we aim to provide updated information for appropriate decision-making to further improve their outcomes.

Stable coronary artery disease (CAD) is a reversible supply-demand mismatch related to ischemia, precipitated by the presence of atherosclerotic plaques within the epicardial coronary arteries. An imbalance can be detected through dynamic electrocardiographic features or reversible perfusion defects during stress testing. Over the past 2 decades, after the introduction of revascularization for CAD, multiple studies have been conducted to understand the role of percutaneous coronary intervention (PCI) and coronary artery bypass grafting (CABG) in improving the endpoints. However, iterative studies and data have shown limited reduction in the rates of myocardial infarction and mortality with revascularization, even in patients with a significant burden of myocardial ischemia. In this review, we present and discuss the contemporary assessment and treatment of patients with stable CAD after the publication of these trials, with particular attention paid to patients in Asian countries, highlighting areas of controversy (Central Illustration).

**Central Illustration:**
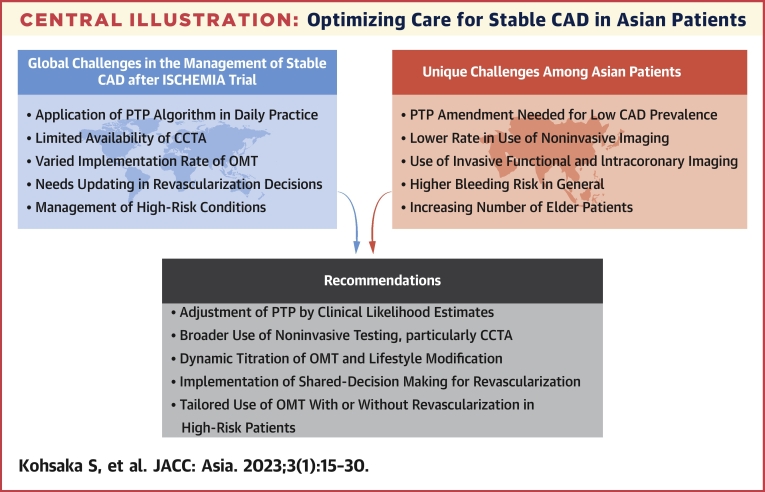
Optimizing Care for Stable CAD in Asian Patients With advances in medical therapy and revascularization techniques, outcomes in patients with stable coronary artery disease (CAD) have progressively improved. However, unmet needs still exist between guideline recommendations and pretest probability (PTP) estimation, application of noninvasive testing, pharmacotherapy, and revascularization in Asian countries. Updated decision making and management in patients with stable CAD, including renewed evidence, should be encouraged. CCTA = coronary computed tomographic angiography; ISCHEMIA = International Study of Comparative Health Effectiveness With Medical and Invasive Approaches; OMT = optimal medical therapy.

## Estimating Pretest Probability

Estimating the likelihood of CAD is universally considered the initial step in the management of patients with stable CAD. However, most patients presenting with chest pain do not have significant coronary lesions. Various systematic algorithms have been proposed to facilitate the diagnostic process.^1^, ^2^, ^3^ In Western countries, the classic Diamond-Forrester approach has long been recommended, although recent observational studies have demonstrated potential overestimation of the likelihood of CAD in the contemporary era.^4^ For example, in the PROMISE (Prospective Multicenter Imaging Study for Evaluation of Chest Pain) trial, the proportion of patients with obstructive CAD was only 13.9% among those classified as “intermediate risk” on the basis of pretest probability (PTP) using the Diamond-Forrester approach.^5^^,^^6^ Therefore, in 2019, the European Society of Cardiology guidelines updated the estimated PTP in the diagnostic algorithm for stable CAD, adjusting for the lower likelihood of CAD. Dyspnea was added to the symptom characteristics.^7^ A similar trend was also reported in the 2021 American Heart Association/American College of Cardiology chest pain guidelines, wherein patients were rarely categorized as high probability (>50%) for CAD in the baseline assessment.^8^ The decrease in CAD prevalence in Western countries may be associated with changes in lifestyle and improvement in preventive medical therapy.

**Figure 1:**
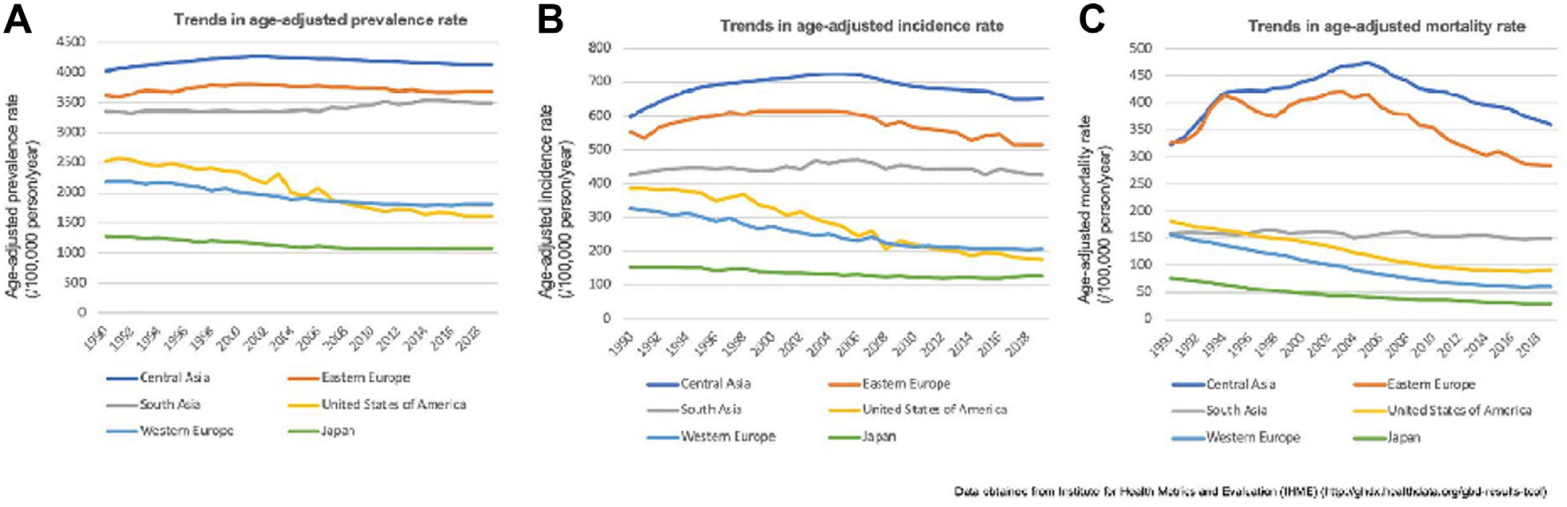
Prevalence, Incidence, and Mortality of Coronary Artery Disease Prevalence **(A)**, incidence **(B)**, and mortality **(C)** of coronary artery disease across the world. Prevalence and incidence remain high in South and Central Asia.

In Asian countries, the incidence of CAD varies among regions. Epidemiologic studies have demonstrated that West, South, and Central Asia have higher and increasing CAD prevalence and mortality than Western countries (Figure 1).^9^, ^10^, ^11^, ^12^, ^13^ Countries in South Asia, including India, Pakistan, and Nepal, are facing an early staged CAD epidemic with an increase in premature CAD deaths, yet the proportion among total deaths is relatively low.^12^ In contrast, the prevalence of CAD in countries in East Asia, such as Japan and Korea, is decreasing and is lower than in other parts of the world.^14^ These geographic differences in CAD prevalence are likely related to the prevalence of risk factors and socioeconomic status in each country.^15^^,^^16^

Given regional variance in the prevalence of CAD in Asian countries, the estimation of PTP using Western algorithms may be inaccurate. PTP should be adjusted on the basis of the regional prevalence of CAD, as discussed earlier. Recently, the Japanese Circulation Society has updated its guidelines for the management of stable CAD.^17^ The use of 2019 European Society of Cardiology PTP as reference data is provisionally recommended in its diagnostic algorithms. However, given the lower prevalence of CAD in Japan, using the European algorithm may lead to unnecessary diagnostic imaging and medical costs.^18^ To better understand the prevalence of CAD in Japan, national epidemiologic surveillance has recently been launched.^19^

PTP should be adjusted by assessing individual clinical likelihood. The clinical likelihood is modified on the basis of traditional risk factors (ie, hypertension) and basic tests (ie, resting electrocardiography, echocardiography, laboratory tests), with an emphasis on left ventricular function and kidney function. A recent study demonstrated that incorporating the coronary artery calcium score into the clinical likelihood assessment can improve the accuracy of CAD diagnosis. In the future, novel prediction tools such as algorithms involving machine learning can be used to improve the diagnosis of CAD in the Asian population.^20^

## Noninvasive Testing

Noninvasive cardiac imaging has advanced significantly over the past few decades. As stated in the previous section, the choice of testing modality is based largely on PTP assessment.^21^^,^^22^ In general, coronary computed tomographic angiography (CTA) is preferred for patients with low to intermediate PTP given the higher negative predictive value, whereas functional testing is preferred for patients with high PTP,^23^ with some alternation on the basis of institutional or regional availability.^24^

### Coronary CTA and coronary artery calcium scoring

**Figure 2:**
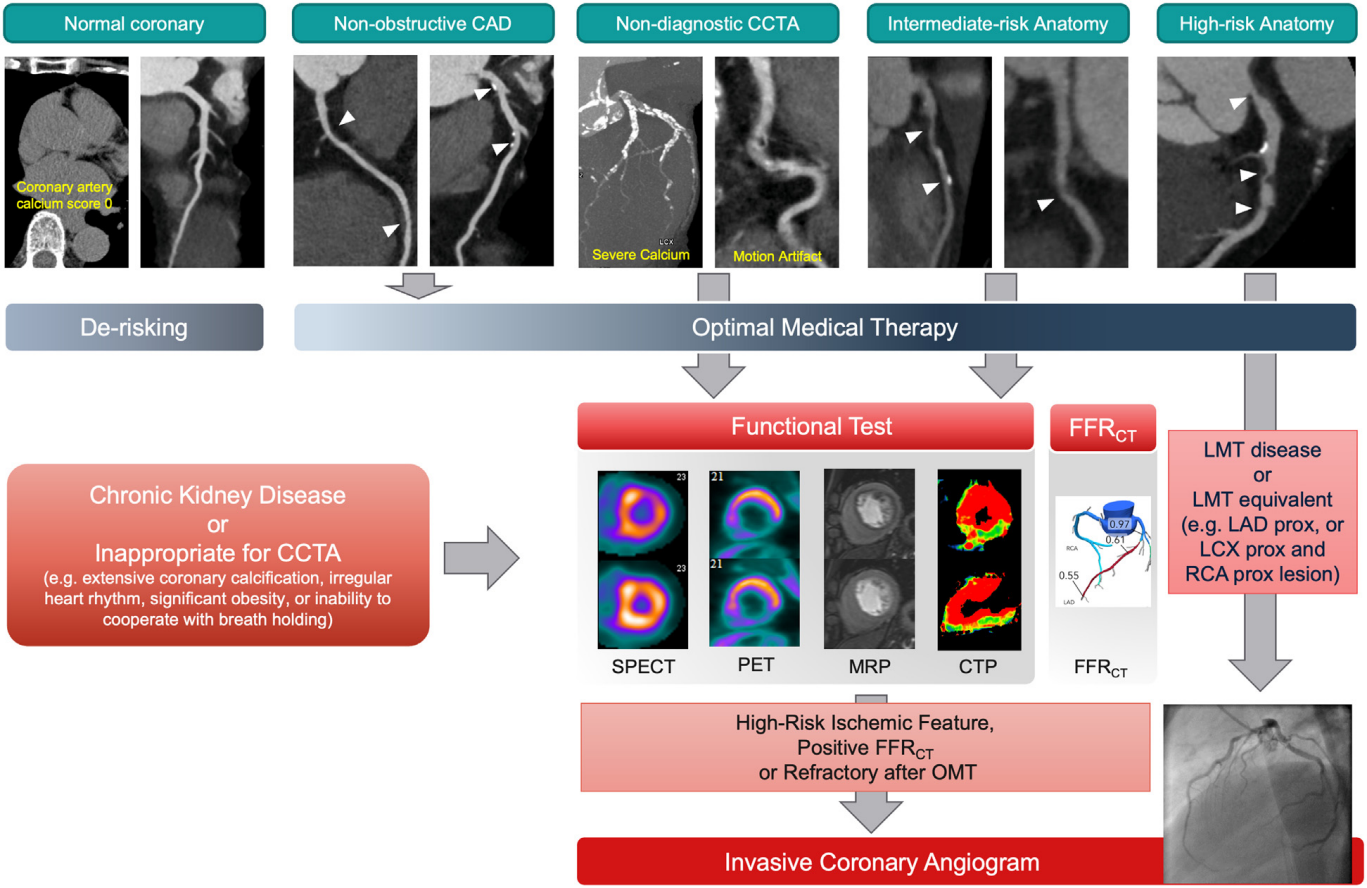
Risk Stratification Using Coronary CTA Patients with zero calcium or normal coronary arteries do not require further testing. Risk factor modification is recommended for all patients with atherosclerotic disease. If coronary computed tomographic angiography (CTA) demonstrates obstructive disease or is nondiagnostic because of severe calcification, further functional testing is recommended. Invasive coronary angiography is necessary when coronary CTA demonstrates left main trunk (LMT) or LMT-equivalent disease. Functional testing can be considered for patients with chronic kidney disease, uncontrollable arrhythmia, heart rate, significant obesity, or inability to cooperate with deep breath-hold. CTP = computed tomographic perfusion; FFR_CT_ = computed tomographic angiography–derived fractional flow reserve; LAD = left anterior descending coronary artery; LCX = left circumflex coronary artery; MRP = magnetic resonance perfusion; OMT = optimal medical therapy; PET = positron emission tomography; prox = proximal; RCA = right coronary artery; SPECT = single-photon emission computed tomography.

Coronary CTA has a high negative predictive value and is increasingly being applied in patients with stable CAD (Figure 2). In both Japanese and European clinical practice guidelines, coronary CTA is recommended as the preferred imaging modality for patients with low to intermediate PTP.^7^^,^^17^ The U.S. clinical practice guidelines also emphasize the role of “guiding” treatment among these patients (Table 1).^8^ The SCOT-HEART (Scottish Computed Tomography of the Heart) trial and the PROMISE study demonstrated that coronary CTA was better at identifying coronary atherosclerosis, leading to higher rates of initiation of optimal medical therapy (OMT) than the control arm.^25^^,^^26^ Furthermore, the Western Denmark Heart Registry demonstrated that a higher atherosclerotic burden visualized using coronary CTA was associated with higher efficacy of statin therapy.^27^

**Table 1:** Recommendations for Use of CT in International Guidelines

	2019 ESC Guideline^11^	2021 AHA/ACC/ASE/CHEST/SAEM/SCCT/SCMR Guideline^10^	2022 JCS Guideline Focused Update^8^
Recommendations for use of coronary CTA in the initial diagnostic management of symptomatic patients with suspected CAD	Noninvasive functional imaging for myocardial ischemia or coronary CTA is recommended as the initial test to diagnose CAD in symptomatic patients in whom obstructive CAD cannot be excluded by clinical assessment alone (Class 1, Level of Evidence: B). Coronary CTA is not recommended when extensive coronary calcification, irregular heart rate, significant obesity, inability to cooperate with breath-hold commands, or any other conditions make obtaining a good image quality unlikely (Class 3, Level of Evidence: C). Risk stratification, preferably using stress imaging or coronary CTA (if permitted by local expertise and availability) or exercise stress ECG (if significant exercise can be performed and ECG is amenable to the identification of ischemic changes), is recommended in patients with suspected or newly diagnosed CAD (Class 1, Level of Evidence: B).	For intermediate- to high-risk patients with stable chest pain and no known CAD, coronary CTA is effective for diagnosis of CAD, risk stratification, and guiding treatment decisions (Class 1, Level of Evidence: A). For patients who have stable chest pain with previous coronary revascularization, coronary CTA is reasonable to evaluate bypass graft or stent patency (for stents ≥3 mm) (Class 2A, Level of Evidence: B-NR). In patients who have had prior CABG surgery presenting with stable chest pain who are suspected to have myocardial ischemia, it is reasonable to perform stress imaging or coronary CTA to evaluate for myocardial ischemia or graft stenosis or occlusion (Class 2A, Level of Evidence: C-LD). For symptomatic patients with known nonobstructive CAD who have stable chest pain, coronary CTA is reasonable for determining atherosclerotic plaque burden and progression to obstructive CAD and guiding therapeutic decision-making (Class 2A, Level of Evidence: B-NR).	Noninvasive anatomical (coronary CTA) or functional imaging test (SPECT, stress CMR, or stress echocardiography) is recommended for the diagnosis of CAD and assessment of event risk in patients with intermediate or high PTP of CAD (Class 1, Level of Evidence: A).
Recommendations for use of coronary CTA as sequential or add-on testing if index test results are positive or inconclusive	Coronary CTA should be considered as an alternative to invasive angiography if other noninvasive test results are equivocal or nondiagnostic (Class 2A, Level of Evidence: C).	For intermediate- to high-risk patients with stable chest pain after inconclusive or abnormal results on exercise ECG or stress imaging, coronary CTA is reasonable (Class 2A, Level of Evidence: B-NR). For intermediate- to high-risk patients with stable chest pain after negative stress test results but with high clinical suspicion of CAD, coronary CTA or ICA may be reasonable (Class 2B, Level of Evidence: C-EO).	
Recommendations for use of CAC testing in the initial diagnostic management of symptomatic patients with suspected CAD	Assessment of CAC score with computed tomography may be considered as a risk modifier in the cardiovascular risk assessment of asymptomatic subjects. (Class 2B, Level of Evidence: B). CAC detection by CT is not recommended to identify individuals with obstructive CAD (Class 3, Level of Evidence: C).	For patients with stable chest pain and no known CAD categorized as low risk, CAC testing is reasonable as a first-line test for excluding calcified plaque and identifying patients with low likelihood of obstructive CAD (Class 2A, Level of Evidence: B-R). For intermediate- to high-risk patients with stable chest pain and no known CAD undergoing stress testing, the addition lof CAC testing can be useful (Class 2A, Level of Evidence: B-NR).	CAC scan or exercise ECG may be considered as an optional test to help rule out CAD in asymptomatic or minimally symptomatic patients with low PTP (Class 2B, Level of Evidence: B).
Recommendations for use of FFR_CT_	NA	For intermediate- to high-risk patients with stable chest pain and known coronary stenosis of 40%-90% in a proximal or middle coronary segment on coronary CTA, FFR_CT_ can be useful for diagnosis of vessel-specific ischemia and to guide decision making regarding the use of coronary revascularization (Class 2A, Level of Evidence: B).	Complementary functional tests (ie, functional imaging tests and FFR_CT_) should be considered for further risk assessment or in patients whose findings on coronary CTA are inconclusive (Class 2A, Level of Evidence: B).

ACC = American College of Cardiology; AHA = American Heart Association; ASE = American Society of Echocardiography; CAC = coronary artery calcium; CAD = coronary artery disease; CMR = cardiac magnetic resonance; CTA = computed tomographic angiography; CHEST = American College of Chest Physicians; CT = computed tomography; ECG = electrocardiogram; ESC = European Society of Cardiology; FFR_CT_ = computed tomography–derived fractional flow reserve; ICA = invasive coronary angiography; JCS = Japanese Circulation Society; NA = not available; PTP = pretest probability; SAEM = Society for Academic Emergency Medicine; SCCT = Society of Cardiovascular Computed Tomography; SCMR = Society for Cardiovascular Magnetic Resonance; SPECT = single-photon emission computed tomography.

Coronary artery calcium scoring has also been shown to have prognostic value, predominantly in asymptomatic subjects (Table 1). Japanese and U.S. guidelines state that ruling out CAD in patients with low PTP and coronary artery calcium scores of zero may be reasonable,^17^ while European guidelines refrain from the use of coronary artery calcium scoring alone to rule out CAD.^7^

### Functional testing

Since the publication of the ISCHEMIA (International Study of Comparative Health Effectiveness With Medical and Invasive Approaches) trial, the role of functional testing in patients with stable CAD has been debated. Although stress electrocardiography is commonly performed given its lower cost, diagnostic accuracy is limited compared with stress imaging. Therefore, current U.S. clinical practice guidelines recommend the use of stress imaging for patients with high PTP (Class 1), patients who cannot undergo coronary CTA, and those with nondiagnostic results (Class 2A).^8^ Functional imaging testing has reasonable diagnostic accuracy in the detection of obstructive CAD (>50% by invasive coronary angiography [ICA]), and the diagnostic accuracy further improves when referenced to invasive fractional flow reserve (FFR) (Supplemental Table 1).

Among the available functional imaging tests, single-photon emission computed tomography is a readily accessible and well-established imaging technique that is routinely used at many institutions.^28^^,^^29^ Compared with coronary CTA and magnetic resonance imaging, the strength of single-photon emission computed tomography includes its use for risk stratification of high-risk patients, including those with chronic kidney disease, and prognostication using perfusion recovery after revascularization.^30^^,^^31^

### Evidence-practice gaps in Asia

Recent studies have shown that noninvasive cardiac tests are a feasible alternative tool for patients with stable CAD. The DISCHARGE (Diagnostic Imaging Strategies for Patients With Stable Chest Pain and Intermediate Risk of Coronary Artery Disease) study showed that the coronary CTA strategy was associated with fewer procedural events, with similar rates of major cardiovascular events compared with the ICA strategy.^21^ The MR-INFORM (Myocardial Perfusion CMR Versus Angiography and FFR to Guide the Management of Patients With Stable Coronary Artery Disease) trial showed that the stress magnetic resonance perfusion strategy was noninferior compared with the FFR strategy for high-risk patients.^22^ Although availability is still limited, the diagnostic accuracy of magnetic resonance perfusion is higher than that of single-photon emission computed tomography because of superior spatial resolution, with better detectability of multivessel disease, comparable with positron emission tomography and computed tomographic perfusion (Supplemental Table 1).

These results provide strong evidence that noninvasive testing should be performed first in the management of patients with stable CAD^17^; however, a recent study indicated that only one-third of patients who underwent PCI had preprocedural noninvasive cardiac testing including coronary CTA and functional tests in Japan, which is significantly lower than reported in Western countries.^32^ In contrast, Japan has the highest number of computed tomographic scanners among the Organisation for Economic Co-operation and Development countries, and the number of coronary CTA cases has rapidly increased over the past 15 years (Supplemental Figure 1).^33^ In fact, the JROAD (Japanese Registry of All Cardiac and Vascular Diseases) showed that the annual number of coronary CTA studies has surpassed the annual number of stress myocardial scintigraphy studies and, recently, the number of invasive coronary angiographic examinations (Supplemental Figure 2).^34^ In contrast, the use of stress perfusion imaging studies, including positron emission tomography, computed tomographic perfusion, and magnetic resonance perfusion, has been limited in Japan because its availability depends on local imaging expertise.^35^

Despite the introduction of coronary CTA, the annual volume of invasive coronary angiographic examinations in Japan has not changed substantially. One possible explanation is the significant shortage of certified radiologists compared with other countries.^36^ A recent survey reported that the number of computed tomographic and magnetic resonance examinations per radiologist was the highest among developed countries, and the potential work load was approximately 4 times that of other countries.^37^ A paradigm shift in the approach to imaging ischemic heart disease is needed, including updated prediction and probability models, a more profound understanding of CAD, and algorithms focused on evaluating both functional and anatomical plaque burden.

## ICA and Revascularization

**Figure 3:**
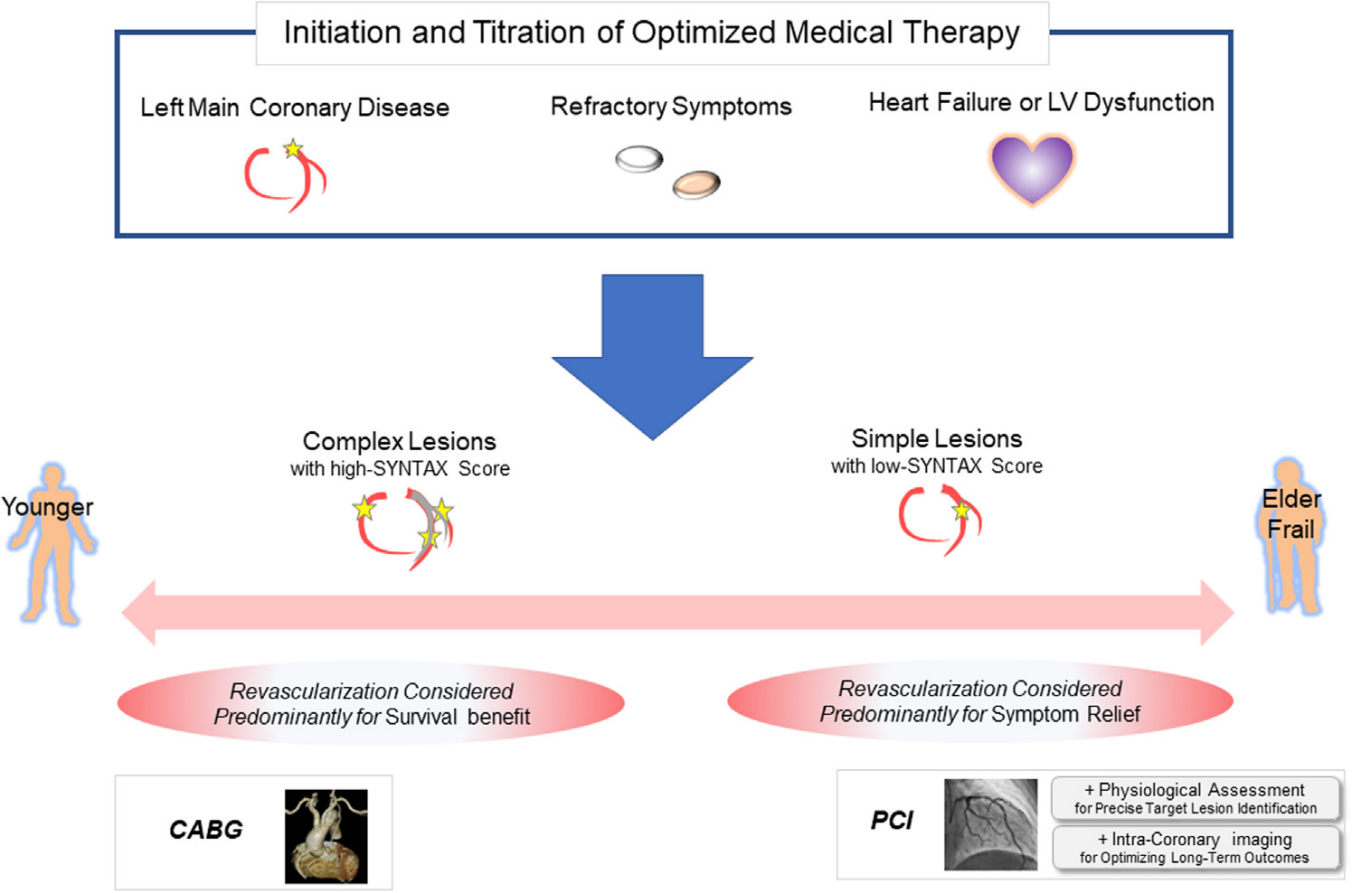
Indication for Revascularization and Selection Between PCI and CABG Medical therapy should be optimized in all patients with chronic coronary artery disease. Invasive coronary angiography is recommended on the basis of the persistence of anginal symptoms or high-risk features by noninvasive testing. CABG = coronary artery bypass grafting; LV = left ventricular; PCI = percutaneous coronary intervention; SYNTAX = Synergy Between PCI With Taxus and Cardiac Surgery.

As described in the previous sections, numerous clinical studies have demonstrated that revascularization procedures ameliorate angina symptoms better than OMT alone, whereas their impact on clinical outcomes is less certain.^38^, ^39^, ^40^, ^41^ Accordingly, the most recent clinical practice guidelines recommend ICA on the basis of the persistence of anginal symptoms or high-risk features by noninvasive testing, indicating the presence of left main coronary disease (Figure 3).^42^

### Functional studies

PCI of functionally significant coronary atherosclerotic lesions assessed via FFR has been shown to reduce major adverse cardiovascular events and ameliorate symptoms compared with OMT alone.^43^ Measurement of FFR requires adenosine administration to minimize distal vascular resistance (linearizing the coronary pressure-flow relationship) and induce hyperemia. More recently, the instantaneous wave-free ratio, which measures the resting pressure gradient across a coronary lesion during diastolic flow (when microvascular resistance is low and stable), has been introduced and can be used in lieu of FFR. At present, 4 angiography-based FFR systems (FFRangio, CathWorks) are available, and each system has individual algorithms to assess the functional severity of coronary stenoses.

However, recent large randomized clinical trials (RCTs) have yielded conflicting results. The FUTURE (Functional Testing Underlying Coronary Revascularization) trial indicated that FFR of all major coronary vessels at the time of diagnostic angiography in patients with multivessel disease did not improve quality of life or angina status following revascularization.^44^ Moreover, in the FAME 3 (Fractional Flow Reserve Versus Angiography for Multivessel Evaluation 3) trial, FFR-guided PCI in patients with 3-vessel disease had a higher primary composite endpoint at 1 year (death, myocardial infarction, stroke, or repeat revascularization) than CABG.^45^ This could be related to the limitations of physiology-guided PCI, where 10% to 30% of vessels remain ischemic by post-PCI FFR assessment, which is associated with adverse clinical outcomes.^46^

### Intracoronary imaging

In addition to the functional assessment of stenotic lesions, the use of intracoronary imaging with intravascular ultrasound (IVUS) or optical coherent tomography (OCT) can also provide additional details such as plaque characteristics or plaque burden that will influence treatment decisions and improve patient outcomes.^47^ A meta-analysis of 31 studies with 17,882 patients showed a reduction in the risk for cardiovascular death and adverse events when using intravascular imaging techniques for PCI guidance.^48^ Furthermore, fast computation of prototype IVUS-based FFR software package (IvusPlus, Pulse Medical Imaging Technology) and OCT-based FFR have become available.^49^^,^^50^ Worldwide, there is vast regional disparity in its use, with the highest uptake in Asia. According to real-world observational studies, IVUS is used in 84.8% and 5.6% of cases in Japan and the United States, respectively.^51^^,^^52^ Interestingly, intracoronary imaging is more frequently used by more experienced operators in Europe and Japan, particularly those with more than 10 years of experience.^53^

OCT has superior resolution compared with IVUS and is suited for detailed assessment (eg, assessment of stents at the strut level or deep calcified plaque morphology). However, image generation by near-infrared light requires blood clearance with contrast injection, which limits the use of OCT in patients with renal insufficiency. Routine use of intracoronary imaging for optimal stenting requires safe manipulation of the imaging catheter and accurate ad hoc image analysis.

### Complete revascularization

As for surgical revascularization, recent observational studies have shown that CABG outcomes have indeed improved over time, with increased use of arterial conduits, advances in stroke prevention strategies (eg, no-touch technique, carotid screening), and increased use of secondary prevention medical therapy.^54^ The achievement of complete revascularization by CABG is an important merit of PCI. A meta-analysis of 89,883 multivessel disease patients reported more frequent incomplete revascularization with PCI than with CABG (56% vs 25%; *P* < 0.001).^55^ The completeness of revascularization has also been emphasized in PCI in recent years and is associated with improved outcomes. Therefore, when evaluating large clinical studies such as ISCHEMIA, it is important to address the extent of completeness of revascularization. Once a PCI-based strategy is chosen, a high success rate of chronic total occlusion PCI is often required to achieve complete revascularization. Patients with concomitant chronic total occlusion and multivessel disease are stratified using the SYNTAX (Synergy Between PCI With Taxus and Cardiac Surgery) score. A hybrid approach with less invasive CABG (eg, totally endoscopic coronary artery bypass) with PCI can also be applied. Collectively, multidisciplinary decision making by the heart team and shared decision making involving patients is essential for tailoring revascularization strategies.

## Optimal Medical Therapy

**Figure 4:**
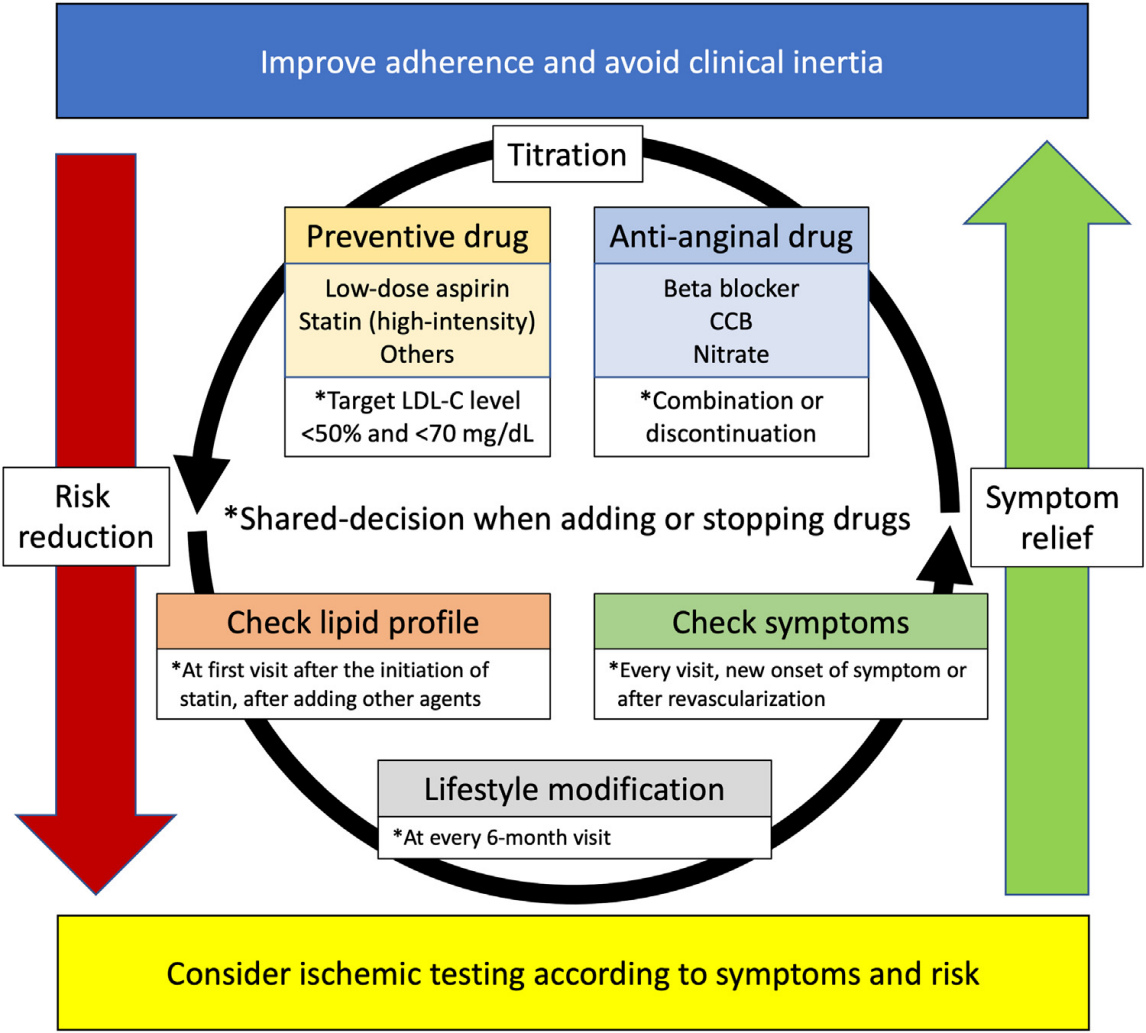
Implementation of Optimal Medical Therapy To improve patient adherence and avoid clinical inertia, continuous risk assessment and corresponding review of optimal medical therapy on the basis of both antianginal and preventive medications are necessary, regardless of revascularization. CCB = calcium-channel blocker; LDL-C = low-density lipoprotein cholesterol.

OMT has been widely considered the initial and preferred approach for the majority of patients with stable CAD. The current clinical guidelines in the United States, Europe, and Japan recommend universal implementation of OMT prior to (and independent of) decision making on revascularization procedures. However, substantial variations in OMT achievement rates remain (Table 2).^38^^,^^39^^,^^56^, ^57^, ^58^ The cause is thought to be multifactorial, and parallel assessment of OMT and long-term prognostic risk is necessary in the contemporary management of stable CAD (Figure 4). In this section, we aim to provide practical recommendations on OMT for Asian patients in accordance with updated findings from large-scale clinical studies.

**Table 2:** Medical Treatment in Patients With Stable CAD Among Clinical Trials and Observational Studies

	COURAGE (Medical Therapy Group at 5 y), 2007^38^	ISCHEMIA (Last Visit in Conservative Group), 2020^39^	NCDR, 2014, United States^56^	T-SPARCLE (CAD-Only Group), 2015, Taiwan^57^	JCD-KiCS (ISCHEMIA-Eligible Group), 2020, Japan^58^
Nitrates	57%	NA	NA	NA	NA
Beta-blockers	86%	NA	78.4%	51.5%	69.1%
Aspirin	94%	96.8%	NA	70.3%	98.7%
Statins	93%	95.3%	73.3%	75.2%	86.8%
High-intensity	NA	65.8%	NA	NA	NA
ACE inhibitors/ARBs	62%/16%	69.7%	75.5%	18.7%/44%	58.8%

ACE = angiotensin-converting enzyme; ARB = angiotensin receptor blocker; CAD = coronary artery disease; COURAGE = Clinical Outcomes Utilizing Revascularization and Aggressive Drug Evaluation; ISCHEMIA = International Study of Comparative Health Effectiveness With Medical and Invasive Approaches; JCD-KiCS = Japan Cardiovascular Database–Keio Interhospital Cardiovascular Studies; NA = not applicable; NCDR = National Cardiovascular Data Registry; T-SPARCLE = Taiwan Secondary Prevention for Patients With Atherosclerotic Disease.

### Antianginal medications

Beta-blockers are the first-line therapy for symptom alleviation by lowering the heart rate and myocardial oxygen demand. Beta-blockers are also known to lower the risk for adverse cardiovascular events in high-risk patients (eg, heart failure or previous myocardial infarction). Conversely, the use of calcium-channel blockers and nitrates has not been shown to be associated with improvement in long-term prognosis but relieves angina symptoms via its vasodilatory effects and is recommended as a second-line therapy. In Asia, however, vasospastic angina is observed rather frequently, and calcium-channel blockers and nitrates are used more frequently than in Western countries.^59^ Patients with epicardial vasospasm are also known to have an increased risk for myocardial infarction.^60^ The prognostic impact of calcium-channel blockers and nitrates in such patients remains unclear and requires further investigation.

### Preventive medications

#### Improving prognosis

Anginal symptoms are typically directly or indirectly caused by impaired coronary flow from stenotic lesions, whereas adverse coronary events are often caused by the sudden rupture (or erosion) of atherosclerotic coronary plaques. Importantly, the risk for plaque rupture is independent of the degree of stenosis.^47^ For plaque stabilization, antiplatelet agents and statins, along with aggressive lifestyle modifications, are recommended.

#### Antithrombotic agents

**Figure 5:**
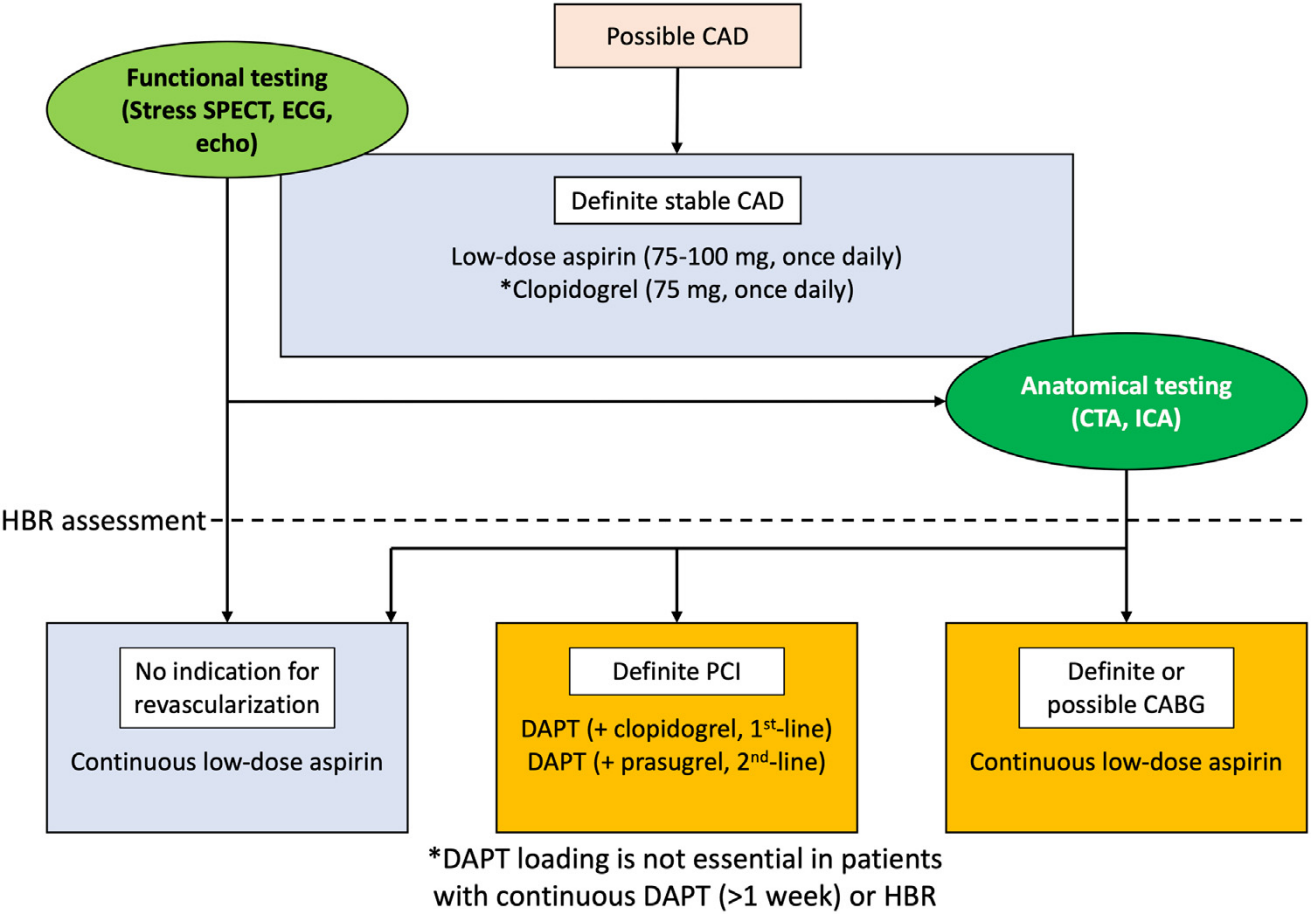
Antiplatelet Use During the Perirevascularization Phase of Stable CAD Low-dose aspirin (75-100 mg once daily) is a primary antithrombotic agent used for secondary prevention of stable CAD. Clopidogrel (75 mg once daily) is an alternative to low-dose aspirin in patients with stable CAD with other vascular comorbidities. After anatomical testing, dual antiplatelet therapy (DAPT) is recommended for patients with planned PCI. ECG = electrocardiography; echo = echocardiography; HBR = high bleeding risk; other abbreviations as in Figures 2 and 3.

Figure 5 shows the perioperative indications for antithrombotic agents in patients with stable CAD. Low-dose aspirin (75-100 mg once daily) is recommended for patients with stable CAD. In patients with histories of other vascular diseases, such as ischemic stroke and peripheral artery disease, clopidogrel (75 mg once daily) is an acceptable alternative. Dual antiplatelet therapy (DAPT) with clopidogrel added to low-dose aspirin is recommended for patients undergoing coronary stent placement. However, the precise preprocedural loading regimen remains unclear. In East Asia, where the risk for bleeding is high, a lower dose of antiplatelet agents is frequently used. For example, prasugrel at a reduced dose (loading 20 mg, maintenance 3.75 mg once daily) is recommended as an alternative to clopidogrel in Japan.^61^

**Figure 6:**
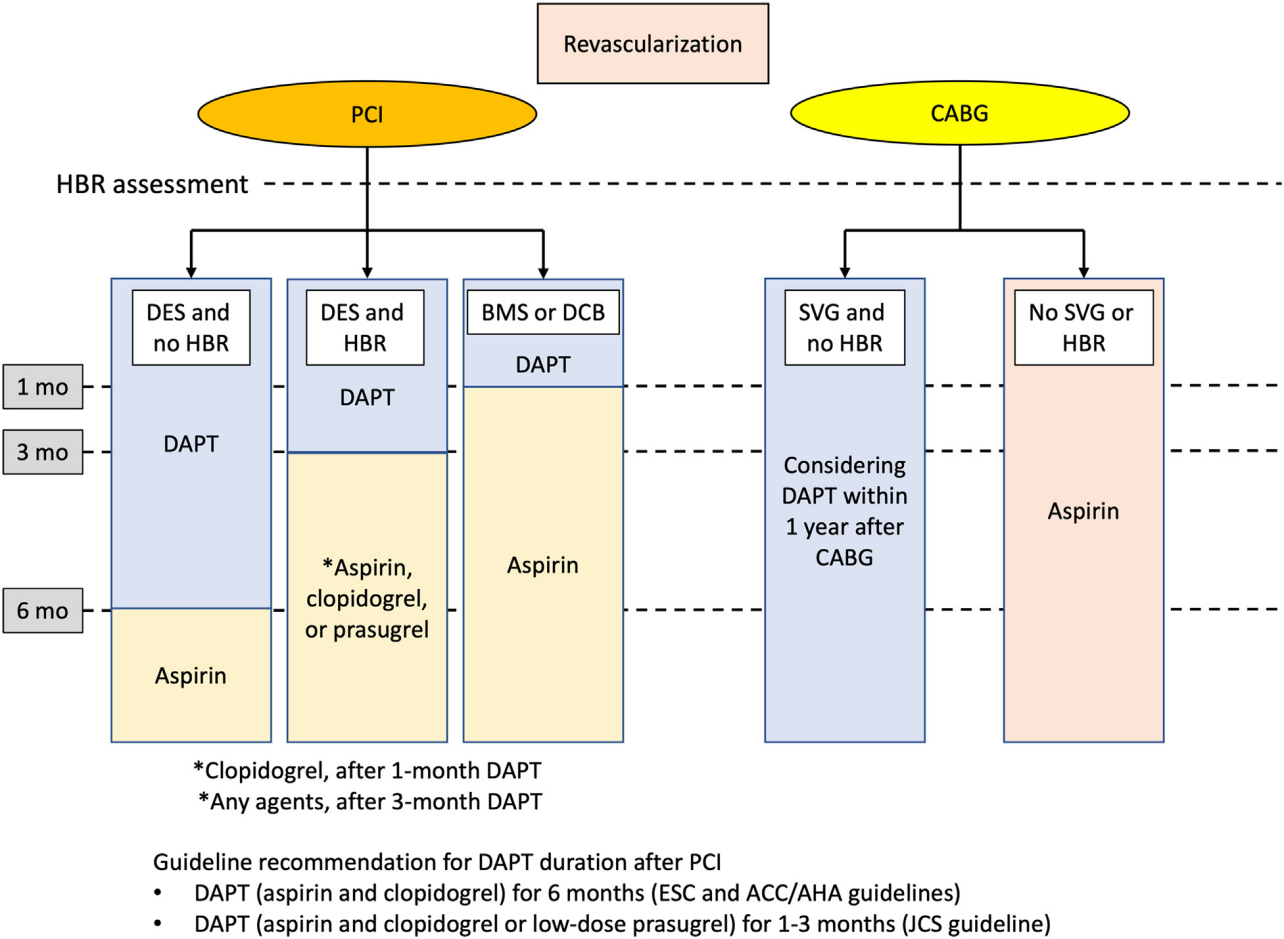
Antiplatelet Use After Revascularization in Patients With Stable CAD Assessment of HBR should be performed during perirevascularization. DAPT duration after PCI depends on the PCI procedure and HBR. DAPT for 1 to 3 months is recommended for patients with HBR after PCI with drug-eluting stents (DES). In patients who undergo PCI with bare-metal stents (BMS) or drug-coated balloons (DCBs), DAPT duration should be 1 month. In post-CABG patients, low-dose aspirin is the primary antiplatelet therapy. DAPT prevents graft thrombosis in patients with saphenous vein graft (SVG) without HBR. European Society of Cardiology (ESC) and American College of Cardiology (ACC)/American Heart Association (AHA) guidelines recommend DAPT (aspirin and clopidogrel) for 6 months. The Japanese Circulation Society (JCS) recommends DAPT (aspirin and clopidogrel or low-dose prasugrel) for 1 to 3 months in patients with stable CAD undergoing PCI. Abbreviations as in Figures 3 and 5.

Figure 6 shows the recommended antiplatelet therapy after revascularization in patients with stable CAD in Asia. Recently, U.S., European, and Asian investigators have jointly published the Academic Research Consortium for High Bleeding Risk criteria.^62^ The updated Japanese clinical practice guidelines recommend an assessment of high bleeding risk, which classifies several risk factors such as low body weight, heart failure, and peripheral artery disease, as high bleeding risk factors are particularly important in Asian populations. In the U.S. and European guidelines, 6 months of DAPT after stent placement is recommended for patients with stable CAD,^63^^,^^64^ whereas the Japanese guidelines recommend an even shorter DAPT interval. As a default strategy, DAPT for 1 to 3 months is acceptable; 6 months of DAPT is reserved for patients who are at high risk for ischemic events. Clinical trials in East Asian countries have repeatedly shown that short-term DAPT (1-3 months) reduces the risk for bleeding without increasing the risk for ischemic events (Table 3). To further advance precision and personalized care, the following DAPT strategies are being investigated in ongoing large-scale clinical trials: 1) clopidogrel for antiplatelet monotherapy after 1-month DAPT, compared with 3-month DAPT; 2) low-dose aspirin, compared with DAPT to prevent graft thrombosis in patients with saphenous vein grafts without high bleeding risk; and 3) 1-month DAPT after PCI in patients on oral anticoagulation, compared with oral anticoagulant agents alone in patients with stable CAD and atrial fibrillation.^65^

**Table 3:** Studies Assessing Shorter DAPT of <6 Months in Patients With Stable Coronary Artery Disease

Study (Year)	DAPT Duration, mo	Countries	Antiplatelet Agent After Shorter DAPT	Primary Endpoint	Main Findings
RESET (2012), n = 2,117^87^	3 vs 12	Korea	Aspirin	Composite of cardiovascular death, MI, stent thrombosis, target vessel revascularization, or bleeding at 1 y	3-mo DAPT was noninferior to 12-m DAPT
OPTIMIZE (2013), n = 3,119^88^	3 vs 12	Brazil	Aspirin	Composite of all-cause death, MI, stroke, or major bleeding at 1 y	3-m DAPT was noninferior to 12-mo DAPT
GLOBAL LEADERS (2018), n = 15,968^89^	1 vs 12	International, mainly Europe	Ticagrelor	Composite of all-cause death or myocardial infarction at 2 y	1-mo DAPT was not superior to the other 12-mo DAPT regimen
STOPDAPT-2 (2019), n = 3,045^90^	1 vs 12	Japan	Clopidogrel	Composite of cardiovascular death, MI, ischemic or hemorrhagic stroke, definite stent thrombosis, or major or minor bleeding at 1 y	1-mo DAPT was noninferior and superior to 12-mo DAPT
SMART-CHOICE (2019), n = 2,993^91^	3 vs 12	Korea	Clopidogrel, prasugrel, or ticagrelor	Composite of all-cause death, MI, or stroke at 1 y	3-mo DAPT was noninferior to 12-mo DAPT
TWILIGHT (2019), n = 9,006^92^	3 vs 12	International, mainly United States	Ticagrelor	Bleeding Academic Research Consortium type 2, 3, or 5 bleeding	3-mo DAPT significantly reduced clinically relevant bleeding than 12-mo DAPT

DAPT = dual antiplatelet therapy; GLOBAL LEADERS = A Clinical Study Comparing Two Forms of Anti-Platelet Therapy After Stent Implantation; MI = myocardial infarction; OPTIMIZE = Optimized Duration of Clopidogrel Therapy Following Treatment With the Endeavor; RESET = Real Safety and Efficacy of 3-Month Dual Antiplatelet Therapy Following Endeavor Zotarolimus-Eluting Stent Implantation; SMART-CHOICE = Comparison Between P2Y_12_ Antagonist Monotherapy and Dual Antiplatelet Therapy After DES; STOPDAPT-2 = Short and Optimal Duration of Dual Antiplatelet Therapy-2 Study; TWILIGHT = Ticagrelor With Aspirin or Alone in High-Risk Patients After Coronary Intervention.

#### Lipid-lowering agents

High-intensity statin therapy is strongly recommended for cardiovascular event reduction and is considered by many experts as the cornerstone of modern stable CAD management. However, substantial regional or institutional variations in its prescription remain.^7^^,^^17^^,^^66^ Lower dose statins continue to be prescribed to older patients with comorbid conditions.^67^ In Asia, many countries have an aging population, and further studies to elucidate the effective dose of statins in elderly patients with CAD are warranted. Current recommendations include titrating statins to the targeted low-density lipoprotein cholesterol level (<50% reduction from baseline and <70 mg/dL). Higher doses of statins have been shown to reduce cardiovascular events in Japanese patients with stable CAD.^68^ If the low-density lipoprotein goal is not achieved even after the initiation of high-dose statin therapy, adding ezetimibe or proprotein convertase subtilisin/kexin type 9 inhibitors is recommended. Fibrate and eicosatetraenoic acid are acceptable for patients with stable CAD with hypertriglyceridemia. As ezetimibe and eicosatetraenoic acid were associated with reduced coronary events in elderly Japanese patients,^69^^,^^70^ these agents may be considered along with statin therapy to stabilize the residual risk in Asian patients with stable CAD.

### Lifestyle modification

In addition to OMT, lifestyle modifications, including exercise, diet modification, and smoking cessation, are fundamental for the secondary prevention of CAD. A previous study showed that smoking cessation reduced fatal CAD events even in smokers.^71^ The age-adjusted prevalence of smoking in Asian countries varies, ranging from 9.6% to 45.5%.^12^ Therefore, smoking cessation is a promising approach for secondary prevention in Asian countries with high smoking prevalence. We recommend the assessment of lifestyle modifications to patients every 6 months (Figure 4).

## High-Risk Populations

Although the results from large-scale RCTs are important to guide clinical practice, RCTs are often limited by stringent patient selection and exclusion of the sicker population. In the final section of this review, we review the specific considerations for patients with anatomically or clinically complex diseases.

### Patients with left main CAD

Patients with left main CAD have been excluded from the key RCTs (COURAGE [Clinical Outcomes Utilizing Revascularization and Aggressive Drug Evaluation], ISCHEMIA, BARI-2D [Bypass Angioplasty Revascularization Investigation in Type 2 Diabetes], FAME II) studying patients with stable CAD.^38^^,^^39^^,^^41^^,^^72^ In contrast, key trials comparing PCI and CABG in patients with left main CAD (EXCEL [Evaluation of XIENCE Versus Coronary Artery Bypass Surgery for Effectiveness of Left Main Revascularization], PRECOMBAT [Premier of Randomized Comparison of Bypass Surgery Versus Angioplasty Using Sirolimus-Eluting Stent in Patients With Left Main Coronary Artery Disease], and NOBLE [Nordic-Baltic-British Left Main Revascularization]) have shown conflicting results.^73^, ^74^, ^75^

The EXCEL trial showed that PCI was noninferior to CABG, whereas the NOBLE trial failed to show the noninferiority of PCI compared with CABG.^73^^,^^75^ The discordant results between these 2 trials may be explained by different methods of patient selection (the NOBLE trial included patients with acute coronary syndrome [18%] and required FFR-based eligibility ≤0.80), as well as differences in the composite endpoints (EXCEL did not include repeat revascularization). A recent patient-level meta-analysis (4 RCTs, n = 4,394) that included the NOBLE and EXCEL trials showed that at 5 years, composite events of all-cause mortality, myocardial infarction, and stroke were comparable, but the rate of repeat revascularization was higher in the PCI group.^76^

European guidelines recommend both PCI and CABG for patients with left main CAD with low anatomical complexity (low SYNTAX score, Class 1A), whereas the U.S. guidelines consider PCI to be an alternative strategy if coronary anatomy is suitable (Class 2A, Level of Evidence: B-R).^63^^,^^64^ Overall, the risks and benefits of each approach should be individually assessed. For example, the upfront risk of CABG may be significant in patients with short life expectancy, and PCI may be a valid option in such cases. Contemporary risk scoring systems, such as SYNTAX II, which uses 7 clinical factors along with the anatomical SYNTAX I score, can be used to estimate the 5-year major adverse cardiovascular events rate for shared decision making.^77^

### Elderly and frail patients

For elderly or frail patients with stable CAD, a personalized approach is important, as indicated by U.S. clinical practice guidelines.^64^ OMT remains a primary therapeutic approach in vulnerable populations, particularly in Asian countries with aging residents. Specifically, their higher bleeding risk should be considered when deciding the duration and dose of antithrombotic agents. PCI is a reasonable choice for the alleviation of angina symptoms in patients with limited life expectancy, contraindications to surgery, or frailty. A subanalysis of the SYNTAX study demonstrated that in patients aged ≥70 years with multivessel and/or left main CAD, PCI and CABG had similar long-term clinical outcomes and quality-of-life improvements.^78^ The SHINANO registry studied 1,923 Japanese patients aged >75 years with multivessel CAD and assessed whether revascularization with PCI is associated with decreased mid-term ischemic events.^79^ The study findings indicate the clinical benefit of revascularization, ideally with complete revascularization, either surgically or percutaneously. However, the completeness of revascularization that needs to be achieved may be different for frail populations and requires further studies.

### Chronic kidney disease and end-stage renal disease

Patients with moderate to severe chronic kidney disease have a 4-fold higher risk for developing multivessel disease, as assessed by coronary angiography, than those with mild or no renal insufficiency, after controlling for diabetes.^80^ ISCHEMIA-CKD is the largest RCT to date examining the effect of PCI for stable CAD in patients with advanced chronic kidney disease (estimated glomerular filtration rate <30 mL/min/1.73 m^2^). At a median follow-up of 2.2 years, an early invasive strategy combined with OMT showed no benefit compared with OMT alone (with revascularization reserved for OMT failure).^81^ Therefore, medical optimization should be further emphasized in patients with chronic kidney disease and end-stage renal disease, reserving ICA for patients with refractory angina symptoms or hypotension during dialysis. When optimizing medical therapy for this patient population, it is important to consider the high bleeding risk from antiplatelet therapy and the less proven benefits of statins.^82^

### Heart failure and reduced left ventricular ejection fraction

In patients with left ventricular systolic dysfunction, ischemia assessment may be less accurate, and the benefits of revascularization for ejection fraction (EF) improvement are less proven. Even in the presence of epicardial coronary disease, mechanisms such as microvascular dysfunction, impaired coronary blood flow reserve, and elevated filling pressure may contribute to myocardial ischemia. Regardless of revascularization strategy, guideline-directed heart failure medications should be initiated and optimized.

In the STICH (Surgical Treatment for Ischemic Heart Failure) extended trial, patients with multivessel disease and left ventricular ejection fractions (LVEFs) ≤35% randomized to CABG plus OMT had significant reductions in all-cause and cardiovascular mortality compared with those receiving OMT alone (59% vs 66%; *P* = 0.004) at 10 years.^83^ In the European guidelines, CABG is recommended in patients with multivessel disease and low EF if surgical risk is acceptable (Class 1B), whereas PCI should be considered on the basis of anatomical complexity and comorbidities such as diabetes (Class IIa, Level of Evidence: C).^63^ In contrast, the U.S. guidelines recommend CABG over PCI if LVEF is <35% (Class 1) or 35% to 50% (Class 2A).^64^ More recently, a subgroup analysis of the ISCHEMIA trial demonstrated that among patients with at least moderate ischemia and heart failure or left ventricular dysfunction (EF 35%-45%), the initial invasive strategy was associated with fewer cardiac events compared with OMT alone.^84^ Notably, this subgroup analysis included only 7.7% of the ISCHEMIA trial cohort, and EF <35% was a major exclusion criterion of the ISCHEMIA study. The REVIVD BCIS2 (Revascularisation for Ischaemic Ventricular Dysfunction) trial is an ongoing, randomized, prospective, multicenter trial that enrolled patients with ischemic cardiomyopathy (EF ≤35% and extensive CAD with at least 4 viable segments).^85^ Viability-guided revascularization may contribute to improving LVEF, but the data are limited and further studies are necessary.^86^

## Conclusions

Stable CAD includes various clinical stages, depending on the symptoms, before or after revascularization, and the presence or absence of comorbidities. Clinicians should evaluate patients’ condition and risk for ischemic or bleeding events, optimize medical therapy at each clinic visit, and consider revascularization procedures through shared decision making. Ideally, the management plan should be tailored to the circumstances of each Asian country. However, evidence regarding stable CAD among Asians remains insufficient. Although further investigation of OMT of stable CAD in the Asian population is needed, large-scale clinical trials for patients with stable CAD are becoming increasingly challenging, given improved clinical outcomes with the implementation of OMT. It is important to appropriately select imaging studies, improve patient adherence to recommended therapies, optimize revascularization outcomes, and personalize care, particularly in high-risk complex patients.

## Funding Support and Author Disclosures

Dr Kohsaka has received speaker fees and consulting fees from Bristol Myers Squibb and Pfizer; and has received institutional research grant support from Novartis and AstraZeneca. Dr Takagi has received speaker fees from HeartFlow Japan GK; and has received consulting fees from HeartFlow. Dr Fukushima has received speaker fees from Nippon-Mediphysics, PDR Pharma, and Pfizer. Dr Nakano has received speaker fees and consulting fees from Pfizer and Ootsuka. Dr Hirai has received speaker fees and consulting fees from Siemens Healthineers, Asahi Intecc, and Zeon Medical; and has received institutional research grant support from Asahi Intecc and Siemens Healthineers. All other authors have reported that they have no relationships relevant to the contents of this paper to disclose.
