# Supplementary material for: Diagnostic and Therapeutic Strategies for Stable Coronary Artery Disease Following the ISCHEMIA Trial
Source: JACC Asia. 2023 Feb 15;3(1):15–30. doi: 10.1016/j.jacasi.2022.10.013 (PMC9982228; doi:10.1016/j.jacasi.2022.10.013)
Supplement: Supplemental Data [file mmc1.docx]

**Supplemental Figure 1:** Temporal trends in the number of cardiac imaging testing in Japan.


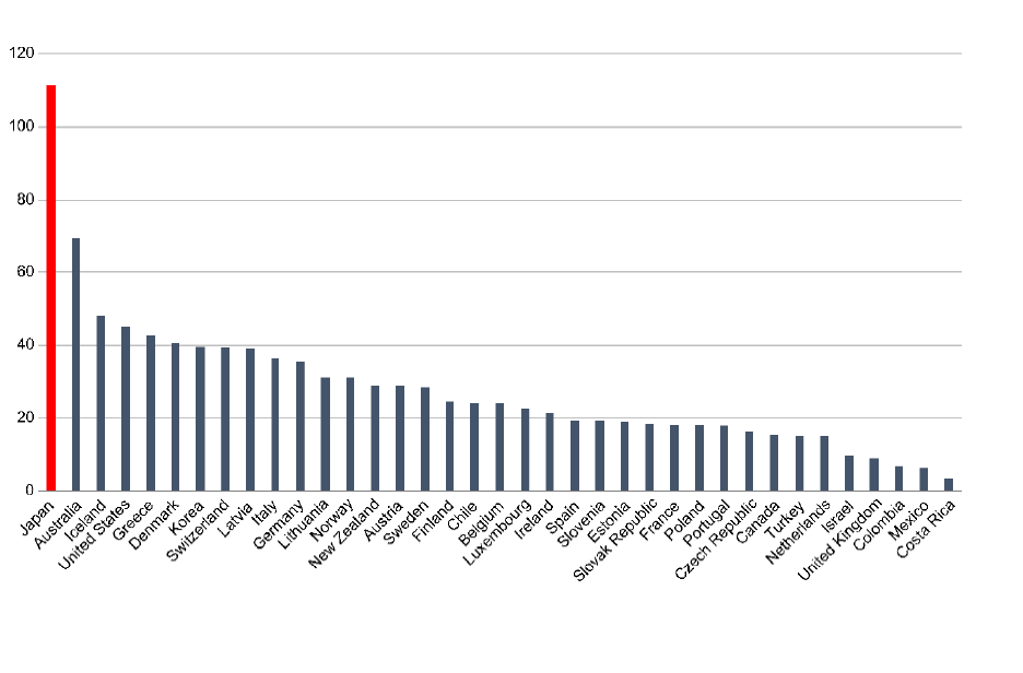


The stacked bars (left Y-axis) represent the annual number of non-invasive cardiac tests. Dotted lines (right Y-axis) depict the annual number of individual cardiac tests.Dotted lines depict the annual number of individual cardiac tests. CCTA, coronary computed tomography angiography; CMR, cardiac magnetic resonance; ECG, electrocardiogram; ICA, invasive coronary angiography; SPECT, single-photon emission computed tomography.

**Supplemental Figure 2:** Number of CT Scanners in OECD countries.


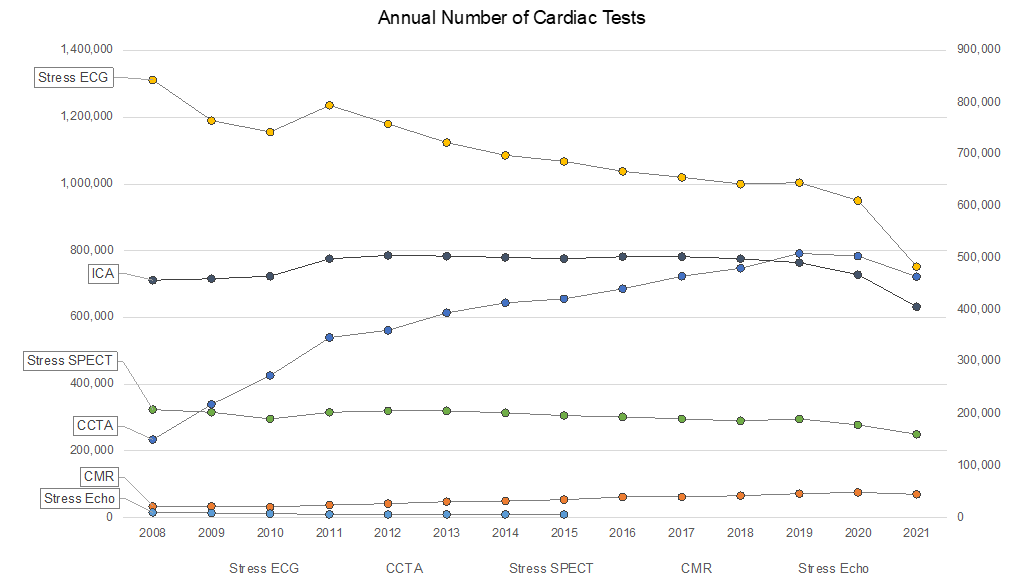


The indicator (Y-axis) indicates the number of CT scanners per 1,000,000 inhabitants. Data from OECD (2022) and CT scanners (indicators). doi:10.1787/bedece12-en (Accessed on May 22, 2022). CT, computed tomography; OECD, organization for economic co-operation and development.

**Supplemental Table 1. Systematic Review and Meta-analysis for Functional Testing in Stable Coronary Artery Disease**

| Referential standards |  | Study/ Author | Journal | Year | n (studies) | Modality | Per-patient or per-vessel diagnostic accuracy |
| --- | --- | --- | --- | --- | --- | --- | --- |
|  |  |  |  |  |  |  |  |
| Angiographical stenosis | ICA(50%) | Nandalur^1^ | J Am Coll Cardiol | 2007 | 2191(37) | Stress CMR, stress MRP | Stress CMR(sens 83%,sepc 86%), stress MRP(sens 91%, spec81%) |
|  | ICA (50%) | Nandalur^2^ | Acad Radiol | 2008 | 1442(19) | PET | Sens 92%, spec 85% |
|  | ICA(50%) | Hamon^3^ | J Cardiovasc Magn Reson | 2010 | 2456(35) | stress MRP | Sens 89%, spec 80% |
|  | CTA(70% or 75%) | de Jong ^4^ | Eur Radiol | 2012 | 2970(28) | stress MRP, SPECT, PET, stress PE | lnDOR for stress MRP(3.63; 95 % CI 3.26–4.00), higher than SPECT and stress PE. SPECT and stress PE were comparable |
|  | CTA(50% or 75%) | Ardle^5^ | J Am Coll Cardiol | 2012 | 3109(23) | PET, SPECT | PET sens 90%, spec 88%; SPECT sens 85%, spec 85% |
|  | ICA (50%) | Jaarsma^6^ | J Am Coll Cardiol | 2012 | 17901(166) | stress MRP, SPECT, PET | Sens SPECT 88%, stress MRP 89%, PET 84%; spec SPECT 61%, stress MRP 76%, PET 81%. |
|  | ICA (50%) | Nielsen^7^ | Eur Heart J Cardiovasc Imaging | 2014 | 216603(7) | CTA, exECG, SPECT | CTA vs exECG sens 98% vs 67%, spec 82% vs 46% |
|  |  |  |  |  |  |  | CTA vs SPECT sens 99% vs 73%, spec71% vs 48% |
|  | CTA(50% or 70%) | Rizvi^8^ | JACC Cardiovasc Img | 2018 | 951(12) | CTA with PET, SPECT, or stress MRP | Per-patient Hybrid vs CTA sens 91% vs 90%, spec 93% vs 66% |
|  |  |  |  |  |  |  | Per-vessel Hybrid vs CTA sens 84% vs 89%, spec 93% vs 83% |
|  |  |  |  |  |  |  |  |
| Functional stenosis | ICA with FFR(0.75 or 0.80) | Danad^9^ | Eur Heart J | 2017 | 3788(23) | CTA, stress MRP, FFRct, SPECT, PET, SE | Per-patient sens CTA 90%, FFRct 90%, MRP 90%, SPECT 70%, SE 77%, ICA 69%, spec CTA 39%, FFRct 71%, MRP 94%, SPECT 78%, SE 75%, ICA 67%. Per-vessel sens CTA 91%, FFRct 83%, MRP 91%, SPECT 57%, ICA 71%, spec CTA 58%, FFRct 78%, MRP 85%, SPECT 75%, SE 75%, ICA 66%. |
|  |  | Takx^10^ | Circ Cardiovasc Imaging | 2015 | 2048(37) | Stress CTP, stress MRP, PET | MRI (NLR, 0.14; 95% CI, 0.10–0.18) CTA (NLR, 0.12; 95% CI, 0.04–0.33) PET ( NLR, 0.14; 95% CI, 0.02–0.87), SPECT (NLR, 0.39; 95% CI, 0.27–0.55) SE (NLR, 0.42; 95% CI, 0.30–0.59). |
|  |  | Li^11^ | JACC Cardiovasc Img | 2014 | 650(14) | stress MRP | Sens 95%, spec 93% |
|  |  | Gonzales^12^ | Am J Cardiol | 2015 | 1535(18) | CTP, FFRct | FFRct (sens 72%, spec 70%), CTP (sens 77%, spec 83%) |
|  |  | Celeng^13^ | JACC Cardiovasc Img | 2019 | 5330(54) | CTP(static or dynamic), FFRct | Per vessel CTP spec86%, FFRct 78% TAG 77%, CTA 61%. CTP, FFRct, and TAG better spec combined with CTA |
|  |  | Pontone^14^ | Int J Cardiol | 2020 | (77) | CTP ,FFRct, PET, SPECT, stress CMR | Sens CTA 88%, FFRct 85%, PET 85%, MRP 81%, CTP with CTA 74%, CTP alone 77%, SE 72%, SPECT 64% |
|  |  |  |  |  |  |  | Spec CTP with CTA 91%, MRP 91%, PET 87% |
|  |  | Dai^15^ | Int J Cardiol | 2016 |  | CTA, PET, CTP ,FFRct, SPECT, PRT, DES | CMR, PET, CTP and FFRCT exhibited comparable sensitivity |
|  |  | Kiaos^16^ | Int J Cardiol | 2018 | 7113(67) | stress MRP | Sens 90%(95%CI, 0.85-0.93), spec 85%(95%CI, 0.80-0.89) |
|  |  | Yang^17^ | Int J Cardiol | 2019 | (28) | stress MRP, SPETC, PET | Sens MRP 88%, SPECT 69%, PET 83% Spec MRP 89%, SPECT 85%, PET 89% |
|  |  | Desai^18^ | Am J Roentgenol | 2013 | 761(12) | stress MRP | Sens 89%, spec 85% |
|  |  | Haberkorn^19^ | Frontiers in Cardiovasc Med | 2021 | 4742(47) | stress MRP, DSE | Sens stress MRP 88%, DSE 72%; spec stress MRP 84%,DSE 89% |
|  |  |  |  |  |  |  |  |
| ICA invasive coronary angiography, MR magnetic resonance imaging, MRP MR perfusion, PET positron emission tomography, SPECT single photon emission computed tomography, CTA computed tomography angiography, CTP CT perfusion, exECG exercise electrocardiogram, FFR fractional flow reserve, SE stress echocardiography, DSE dobutamine SE, PE perfusion echo, CI Confidence Interval, Sens sensitivity, Spec specificity, NLR negative likelihood ratio, InDOR the natural logarithms of the diagnostic odds ratio | | | | | | | |

**References**

1. Nandalur KR, Dwamena BA, Choudhri AF, Nandalur MR, Carlos RC. Diagnostic Performance of Stress Cardiac Magnetic Resonance Imaging in the Detection of Coronary Artery Disease. Journal of the American College of Cardiology 2007;50:1343-1353.

2. Nandalur KR, Dwamena BA, Choudhri AF, Nandalur SR, Reddy P, Carlos RC. Diagnostic Performance of Positron Emission Tomography in the Detection of Coronary Artery Disease: A Meta-analysis. Academic Radiology 2008;15:444-451.

3. Hamon M, Fau G, Née G, Ehtisham J, Morello R, Hamon M. Meta-analysis of the diagnostic performance of stress perfusion cardiovascular magnetic resonance for detection of coronary artery disease. Journal of Cardiovascular Magnetic Resonance 2010;12:29.

4. de Jong MC, Genders TSS, van Geuns R-J, Moelker A, Hunink MGM. Diagnostic performance of stress myocardial perfusion imaging for coronary artery disease: a systematic review and meta-analysis. Eur Radiol 2012;22:1881-1895.

5. Ardle BAM, Dowsley TF, deKemp RA, Wells GA, Beanlands RS. Does Rubidium-82 PET Have Superior Accuracy to SPECT Perfusion Imaging for the Diagnosis of Obstructive Coronary Disease? Journal of the American College of Cardiology 2012;60:1828-1837.

6. Jaarsma C, Leiner T, Bekkers SC et al. Diagnostic Performance of Noninvasive Myocardial Perfusion Imaging Using Single-Photon Emission Computed Tomography, Cardiac Magnetic Resonance, and Positron Emission Tomography Imaging for the Detection of Obstructive Coronary Artery Disease. Journal of the American College of Cardiology 2012;59:1719-1728.

7. Nielsen LH, Ortner N, Nørgaard BL, Achenbach S, Leipsic J, Abdulla J. The diagnostic accuracy and outcomes after coronary computed tomography angiography vs. conventional functional testing in patients with stable angina pectoris: a systematic review and meta-analysis. European Heart Journal - Cardiovascular Imaging 2014;15:961-971.

8. Rizvi A, Han D, Danad I et al. Diagnostic Performance of Hybrid Cardiac Imaging Methods for Assessment of Obstructive Coronary Artery Disease Compared With Stand-Alone Coronary Computed Tomography Angiography: A Meta-Analysis. JACC: Cardiovascular Imaging 2018;11:589-599.

9. Danad I, Szymonifka J, Twisk JWR et al. Diagnostic performance of cardiac imaging methods to diagnose ischaemia-causing coronary artery disease when directly compared with fractional flow reserve as a reference standard: a meta-analysis. European Heart Journal 2016;38:991-998.

10. Takx RAP, Blomberg BA, Aidi HE et al. Diagnostic Accuracy of Stress Myocardial Perfusion Imaging Compared to Invasive Coronary Angiography With Fractional Flow Reserve Meta-Analysis. Circulation: Cardiovascular Imaging 2015;8:e002666.

11. Li M, Zhou T, Yang L-f, Peng Z-h, Ding J, Sun G. Diagnostic Accuracy of Myocardial Magnetic Resonance Perfusion to Diagnose Ischemic Stenosis With Fractional Flow Reserve as Reference. JACC: Cardiovascular Imaging 2014;7:1098-1105.

12. Gonzalez JA, Lipinski MJ, Flors L, Shaw PW, Kramer CM, Salerno M. Meta-Analysis of Diagnostic Performance of Coronary Computed Tomography Angiography, Computed Tomography Perfusion, and Computed Tomography-Fractional Flow Reserve in Functional Myocardial Ischemia Assessment Versus Invasive Fractional Flow Reserve. The American Journal of Cardiology 2015;116:1469-1478.

13. Celeng C, Leiner T, Maurovich-Horvat P et al. Anatomical and Functional Computed Tomography for Diagnosing Hemodynamically Significant Coronary Artery Disease: A Meta-Analysis. JACC: Cardiovascular Imaging 2019;12:1316-1325.

14. Pontone G, Guaricci AI, Palmer SC et al. Diagnostic performance of non-invasive imaging for stable coronary artery disease: A meta-analysis. International Journal of Cardiology 2020;300:276-281.

15. Dai N, Zhang X, Zhang Y et al. Enhanced diagnostic utility achieved by myocardial blood analysis: A meta-analysis of noninvasive cardiac imaging in the detection of functional coronary artery disease. International Journal of Cardiology 2016;221:665-673.

16. Kiaos A, Tziatzios I, Hadjimiltiades S, Karvounis C, Karamitsos TD. Diagnostic performance of stress perfusion cardiac magnetic resonance for the detection of coronary artery disease: A systematic review and meta-analysis. International Journal of Cardiology 2018;252:229-233.

17. Yang K, Yu S-q, Lu M-j, Zhao S-h. Comparison of diagnostic accuracy of stress myocardial perfusion imaging for detecting hemodynamically significant coronary artery disease between cardiac magnetic resonance and nuclear medical imaging: A meta-analysis. International Journal of Cardiology 2019;293:278-285.

18. Desai RR, Jha S. Diagnostic performance of cardiac stress perfusion MRI in the detection of coronary artery disease using fractional flow reserve as the reference standard: a meta-analysis. AJR Am J Roentgenol 2013;201:W245-52.

19. Haberkorn SM, Haberkorn SI, Bönner F, Kelm M, Hopkin G, Petersen SE. Vasodilator Myocardial Perfusion Cardiac Magnetic Resonance Imaging Is Superior to Dobutamine Stress Echocardiography in the Detection of Relevant Coronary Artery Stenosis: A Systematic Review and Meta-Analysis on Their Diagnostic Accuracy. Frontiers in Cardiovascular Medicine 2021;8.
